# Interleukin-6 Modulation in Ovarian Cancer Necessitates a Targeted Strategy: From the Approved to Emerging Therapies

**DOI:** 10.3390/cancers16244187

**Published:** 2024-12-16

**Authors:** Hina Amer, Nirmala C. Kampan, Catherine Itsiopoulos, Katie L. Flanagan, Clare L. Scott, Apriliana E. R. Kartikasari, Magdalena Plebanski

**Affiliations:** 1 School of Health and Biomedical Sciences, RMIT University, Bundoora, VIC 3082, Australia; hinaamer.ha@gmail.com (H.A.); april.kartikasari@rmit.edu.au (A.E.R.K.); 2 Department of Obstetrics and Gynecology, Faculty of Medicine, University Kebangsaan Malaysia, Kuala Lumpur 56000, Malaysia; 3 School of Medicine and School of Health Sciences, University of Tasmania, Launceston, TAS 7250, Australia; 4 Tasmanian Vaccine Trial Centre, Clifford Craig Foundation, Launceston General Hospital, Launceston, TAS 7250, Australia; 5 The Walter and Eliza Hall Institute of Medical Research, Parkville, VIC 3052, Australia; 6 Faculty of Medicine, Dentistry, and Health Sciences, The University of Melbourne, Parkville, VIC 3052, Australia; 7 The Royal Women’s Hospital, Parkville, VIC 3052, Australia

**Keywords:** ovarian cancer, interleukin-6, inflammation, immunotherapy, approved therapy, signaling, small molecules, targeted therapy, clinical trials

## Abstract

Despite significant advances in treatments, ovarian cancer (OC) remains one of the most prevalent and lethal gynecological cancers in women. The frequent detection at the advanced stages has contributed to low survival rates, resistance to various treatments, and disease recurrence. Thus, a more effective approach is warranted to combat OC. The cytokine Interleukin-6 (IL6) has been implicated in various stages of OC development. High IL6 levels are also correlated with a lower survival rate in OC patients. In this current review, we summarized the pivotal roles of IL6 in OC, including the initiation, development, invasion, metastasis, and drug resistance mechanisms. This article systematically highlights how targeting IL6 improves OC outcomes by altering various cancer processes and reports the ongoing clinical trials that would further shape the IL6-based targeted therapies. This review also suggests how combining IL6-targeted therapies with other therapeutic strategies could further enhance their efficacy to combat OC.

## Simple Summary

In this article, we review the role of Interleukin-6 in every stage of ovarian cancer development, highlight the approved and emerging therapies to target Interleukin-6 and its signaling in cancer, and identify their combination with other therapies to provide a more effective and personalized treatment for ovarian cancer.

## 1. Introduction

Ovarian cancer (OC) is one of the most common and lethal gynecological cancers, with a five-year survival rate of less than 48% in Australian women [1]. The Federation of Gynecology and Obstetrics (FIGO) classified OC into four disease stages: Stages I, II, III, and IV, based on the location and extent of the spread of the cancer [2]. Stages I and II are considered early stages (ES) and confined to one or both ovaries and the pelvis. Stages III and IV are late stages (LS), diagnosed in 70% of OC patients, characterized by cancer cells invading the abdomen and distinct metastasis, respectively [3].

Inflammation has emerged as a crucial factor in promoting cancer development [4,5,6]. OC is more commonly developed in old age when the body’s immune system is challenged with chronic inflammatory processes known as inflammaging (inflammation due to aging) and immunosenescent (aging of the immune system) [7,8]. Moreover, obesity, prolonged benign conditions (polycystic ovaries, endometriosis), and recurrent pelvic infections also increase the risk of developing OC [7,9].

Among the various pro-inflammatory cytokines implicated in cancer development, Interleukin-6 (IL6) has gained significant attention due to its multifaceted role in tumorigenesis. This pro-inflammatory cytokine is vital in various physiological processes, including immune cell activation, hematopoiesis, and acute Phase reactions. However, excessive production of IL6 can contribute to the pathogenesis of autoimmune diseases and cancer [4,5,6]. Indeed, recent studies have suggested IL6 as a crucial driver in promoting the development and progression of various cancers, including breast, lung, colon, and ovarian cancer [10,11,12,13].

This review aims to delve into the pathophysiological implications of IL6 in OC. We review the intricate mechanisms by which IL6 influences tumor initiation, growth, metastasis, and resistance to therapy. We discuss the potential utility of targeting IL6 alone and in combination with other drugs to enhance the efficacy of OC treatment. We further report the current approved and trialed IL6-based therapies for OC. By identifying the precise roles of IL6 in OC pathogenesis, summarizing the IL6-based targeted therapies, and discussing the potential of these therapies to be combined with other therapies, we can pave the way for more effective treatment strategies to combat OC and improve patient outcomes.

## 2. IL6 and Its Role in Ovarian Cancer

### 2.1. IL6 Is a Significant Inducer of Inflammation via Its Signaling Pathways, Influencing Immune and Non-Immune Cells and Promoting Subsequent Development of OC Within the Tumor Microenvironment (TME)

**Figure 1:**
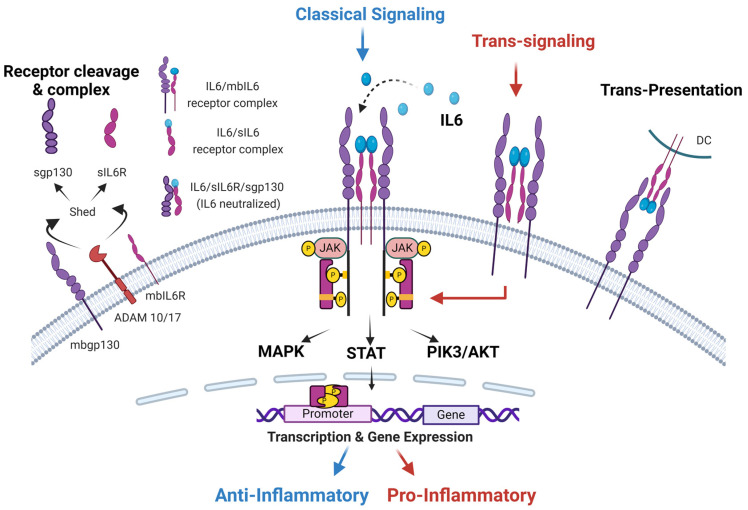
**IL6 classical and trans-signaling activation pathways.** Membrane-bound IL6 receptors (mbIL6R) are present in immune cells. mbIL6R are cleaved by A Disintegrin and Metalloproteinases-10 (ADAM10) or ADAM17 and shed as soluble IL6 receptors (sIL6R) shedding of mbIL6R. Membrane-bound Glycoprotein-130 (mbGp130) is ubiquitously expressed by all cells. Soluble Gp-130 (sGp130) is formed by shedding of mbGp130. IL6 binds to (1) mbIL6R to activate the classical signaling pathway, (2) sIL6R to initiate the trans-signaling pathway, or (3) trans-presented by dendritic cells (DCs) through mbIL6R. Classical signaling and trans-signaling initiate the same signaling cascade; however, due to diversity in cellular presentation, they have an anti-inflammatory or pro-inflammatory effect, respectively [16].

IL6 is a member of the IL6 family of cytokines, which also includes Interleukin 11 (IL11), Interleukin 27 (IL27), Interleukin 31 (IL31), Cardiotrophin 1 (CT1), Cardiotrophin-Like Cytokine Factor 1 (CLCF1), Ciliary Neurotrophic Factor (CNTF), Leukemia Inhibitory Factor (LIF), Neuropoietin (NPN), and Oncostatin M (OSM) [14]. These cytokines share similar structural and functional characteristics. The functional redundancy of the IL6 cytokine family stems from their structural similarity and receptor engagement with a Glycoprotein 130 (Gp130), which contains critical motifs and domains for intracellular signaling (Figure 1) [15].

IL6 can recognize and bind to two distinct receptors, initiating two separate signal transmission pathways (Figure 1) [17]. The first receptor is the membrane-bound IL6 receptor (mbIL6R), an 80kD α-chain protein [18]. These receptors are expressed in a limited range of cell types, including specific immune cells such as T cells, neutrophils, monocytes, megakaryocytes, hepatocytes, and endothelial cells. Upon binding these receptors, the IL6/mbIL6R complex activates Glycoprotein-130 (Gp130) and initiates an anti-inflammatory “classical signaling pathway” through Janus Kinase/Signal Transducer and Activator of Transcription (JAK/STAT) [19]. This activation leads to various targeted physiological functions, such as stimulating Acute Phase Protein (APP) production in response to inflammation in healing processes [20].

The second receptor for IL6 is found in circulation and referred to as the soluble IL6 receptor (sIL6R). These receptors are 50–55kD protein molecules lacking cytoplasmic extension and are present at plasma levels of approximately 25–35 ng/mL in healthy individuals [21]. The formation of sIL6R occurs through alternating mRNA splicing (10% of cases) and predominantly by proteolytic cleavage and ectodermal shedding (90% of cases) of mbIL6R via proteases such as A Disintegrin And Metalloproteinase-10 (ADAM10) or ADAM17 [16]. The IL6/sIL6R complex activates Gp130 receptors, activating the pro-inflammatory “trans-signaling pathway” (Figure 1). This pathway plays a significant role in hematopoiesis, neuron survival, osteoclast proliferation, and the stimulation and activation of endothelial and smooth muscle cells [21]. However, it is also associated with chronic inflammation and triggers the proliferation of cancer cells (Table 1) [22].

IL6 plays a crucial role in immune responses through its unique “trans-presentation signaling mechanism.” The IL6 binds to its receptor (IL6R) on the surface of the dendritic and is displayed to T cells. This direct presentation interacts with gp130 to precisely influence T cell activation and differentiation [33]. Depending on the surrounding cytokine environment, IL6 trans-presentation can promote either pro-inflammatory or anti-inflammatory responses. In a pro-inflammatory setting, along with transforming growth factor beta (TGFβ), IL6 drives the differentiation of Th17 cells, contributing to chronic inflammation and autoimmune conditions [34,35]. In contrast, in the presence of anti-inflammatory cytokines like IL10, IL6 can promote the generation of regulatory T cells (Tregs), thus regulating Treg/Th17 balance [34,36,37]. Therefore, IL6 trans-presentation by dendritic cells plays a vital role in shaping immune responses, balancing inflammation, and influencing the progression of diseases like cancer.

The role of inflammatory cells in the cancer TME was first proposed by Rudolf Virchow in 1863. Virchow highlighted the predominance of lymphocytes within the infiltrate around cancerous tissue and suggested their involvement in the uncontrolled proliferation of cancer [38]. He described that, in the presence of a possible initiative genetic spark, the infiltration in TME would fuel their cellular signaling pathways, aid in accelerating tumor growth, and then spread into neighboring and distant environments [38]. The role of inflammation in cancer progression, particularly the involvement of pro-inflammatory cytokines such as IL6, was widely accepted based on Virchow’s proposal.

The ovarian cancer tumor microenvironment (OC-TME) presents a specialized group of cells that orchestrate the build of an immunosuppressive microenvironment to favor tumor proliferation, cultivate metastasis pathways, shapes to escape immunosurveillance and learn adaptive strategies to develop therapeutic resistance [39]. Ovarian cancer cells within the OC-TME are direct producers of IL6, which they use in an autocrine manner to sustain their growth and survival [40]. IL6 production dramatically increases due to chronically elicited inflammatory responses by aberrantly acting innate and adaptive immune cells and specialized transformed non-immune cells within the OC-TME. Activating other TME components amplifies inflammation and creates a more permissive environment for tumor expansion [41].

The cellular component of the innate immune system produces IL6 within the OC-TME, which plays a crucial role in sustaining a pro-tumorigenic environment, supporting immune evasion, and aiding tumor survival and metastasis. For instance, cancer-associated fibroblast (CAF) produces IL6 within OC-TME, aids in the remolding of the extracellular matrix, and induces epithelial-mesenchymal transition (EMT) [42]. IL6 derived from CAFs creates a positive feedback loop, perpetuating CAF activation to support tumor cell proliferation and survival and stimulate other immune cells in the TME to facilitate invasion and metastasis [43]. The tumor-associated macrophages (TAMs) in OC-TME are often polarized towards an M2-like, pro-tumorigenic phenotype under the effect of elevated IL6 levels and secrete more IL6 to maintain their immunosuppressive function, aiding in immune evasion and promoting tumor progression [44]. Myeloid-derived suppressor cells (MDSCs), another source of IL6 in the TME, support immune suppression by inhibiting T cell function, allowing tumor cells to evade immune responses and increase tumor growth [45]. Although a minor contributor within the OC-TME, endothelial cells can also produce IL6 in response to hypoxia or signals from tumor cells [46]. Along with vascular endothelial growth factor (VEGF), they promote angiogenesis to enhance blood supply, support tumor growth, and facilitate metastasis [47].

The dendritic cells (DCs) in the innate immune arm presents antigens to T cells to establish an anti-tumor response [48]. In OC-TME, this function is impaired via IL6/gp130/STAT3 signaling, leading to irregular DC expression, decreased migration by inhibited CCR7 expression on DC cells, MHCII (major histocompatibility class II) suppression, and impaired T cell activation. Additionally, DCs secrete IL6 in a paracrine manner to maintain its immature form [49,50]. IL6, through the STAT3 pathway, also compromises the function of natural killer (NK) cells by inducing SHP-2, which hinders granule release and reduces their cytotoxic function [51]. The IL6-STAT3 pathway has been shown to enhance neutrophil trafficking, survival, and anti-apoptotic response [52]. In ovarian cancer, studies highlighted the association of IL6 with a high neutrophilic/lymphocytic ratio, which is indicative of poor prognostic factors [53].

To further enhance tumor-supportive conditions in OC-TME, IL6 suppresses adaptive immunity by promoting T cell exhaustion and skewing T cell differentiation [54]. In CD8⁺ T cells, IL6 upregulates immune checkpoints like PD-1, diminishing cytotoxic function and reducing the production of key cytokines, such as interferon-gamma (IFN-γ), thereby impairing anti-tumor responses [55]. In CD4⁺ T cells, IL6 favors differentiation of Th2 cells over Th1 and induces Th17 production, contributing to autoimmunity, chronic inflammation, and supporting tumor growth [56]. IL6 also enhances the expansion of regulatory T cells (Treg) in the presence of transforming growth factor beta (TGF-β), further suppressing T cell activity and promoting immune evasion [57]. Recent research has shown that pre-treatment levels of IL6 and tumor necrosis factor receptor type-2 (TNFR2) expressing Tregs in peripheral blood can effectively differentiate between malignant and benign ovarian masses, and STAT3 signaling pathways potentially drive the interaction between IL6 and TNFR2^+^ Tregs [10].

The complex interplay of IL6 signaling across multiple cell types makes it a promising therapeutic target in OC, offering the potential for strategies to disrupt the tumor-supportive microenvironment and enhance the efficacy of current treatments.

### 2.2. IL6 Accelerates the Cell Cycle, Promotes the Survival of Cancer Cells by Negating Apoptosis, and Induces Cancer Stem Cell Generation

**Figure 2:**
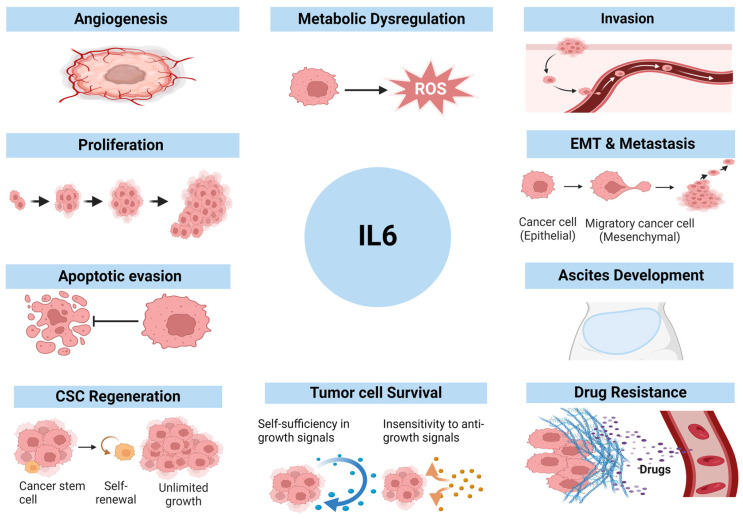
**IL6 promotes cancer progression, invasion, metastasis, and drug resistance.** Aberrant and excessive production of IL6 in cancers can lead to various cancer events, such as metabolic changes as well as cancer invasion, metastasis, and recurrence. EMT, epithelial-mesenchymal transition; CSCs, cancer stem cells; ROSs, reactive oxygen species.

IL6 enhances cell cycle progression in various cells, particularly cancer cells, by activating gene expression for cell growth, proliferation, survival, and differentiation (Figure 2, Table 1) [58]. IL6 activates JAK/STAT and Nuclear Factor-κB (NFκB) pathways and upregulates the cell cycle-promoting proteins such as Cyclin D1, D2, B1, and cMYC [40]. The Cyclin proteins bind and activate Cyclin-Dependent Kinases (CDK), including CDK4 and CDK6, promoting the cell-cycle transition from G1 to the S Phase [59]. These activated CDKs, in turn, phosphorylate and inactivate Retinoblastoma protein (Rb), allowing transcription of genes required for DNA synthesis [60]. Meanwhile, P21 and P27, the negative cell cycle regulators, can bind to CDKs, preventing the phosphorylation of Rb and blocking entry to the S Phase [60]. IL6 downregulates the CDK inhibitors, P21 and P27, facilitating cell entry into the cell cycle and promoting cell proliferation [41,58].

Apoptosis is a programmed cell death triggered by external signals such as death receptors or internal signals from DNA-damaged or oxidative-stressed environments. Evasion of apoptosis is crucial in cancers as it favors uncontrolled development of their desired progeny. In cancer cells, IL6 via JAK/STAT, NFκB, Phosphoinositide-3 Kinase/Protein Kinase B (PI3K/AKT), and Mitogen-Activated Protein Kinase/Extracellular Signal-Regulated Kinase (MAPK/ERK) pathways upregulates anti-apoptotic proteins such as B Cell Leukemia/Lymphoma-2 protein (BCL2) and Survivin, thus inhibiting proapoptotic activation [61,62,63].

Cancer stem cells (CSCs) are spherical-shaped tumor-initiating cells that exist in a small population in TME. Due to their self-renewal, differentiation, and proliferation capability, CSCs are highly tumorigenic, metastatic, and chemo-resistant cells [64]. IL6 can rapidly convert the non-stem cancer cell population to CSCs, as evident in various cancers such as breast, prostate, and bladder cancers [65,66]. Studies in lung cancers highlighted that IL6 protects cancer stem cells (CSCs) (Figure 2, Table 1) [67].

IL6-JAK/STAT pathway upregulates CSC-associated *NANOG* and *Octamer-Binding Transcription Factor-4* (*OCT4*) gene expression, enhancing stemness and malignancy of cancer cells (Figure 2, Table 1) [68]. In OC, IL6 via JAK/STAT promotes the expression of CD44, also known as Homing Cell Adhesion Molecule (HCAM), a transmembrane glycoprotein associated with cancer cell stemness [69,70,71]. CD44 upregulates the Multidrug Resistance Marker (MDR) by forming the STAT3/NANOG complex. It activates the catalytic subunit of telomerase, human Telomerase Reverse Transcriptase (hTERT), which is associated with epithelial-mesenchymal transition (EMT) stimulation, metastasis, drug resistance, disease recurrence, and poor prognosis in OC patients [69,70,71].

### 2.3. IL6 Induces Angiogenesis and OC Invasiveness and Spread

Angiogenesis is the process of the emergence of new blood vessels from pre-existing vascular structures. It is a crucial process for tumor survival, as these vessels establish a continued eternalized paracrine loop that serves as a self-nourishment and waste removal portal in OC-TME and provides a significant route for metastasis [72]. In the OC-TME, IL6 induces angiogenesis in both autocrine and paracrine manners by upregulating vital proangiogenic factors, such as VEGF [58,73]. IL6 causes an increase in the release of chemokines such as CCL2, CXCL12, and Macrophage Migratory Inhibitory Factor (MIF), which are involved in angiogenesis by stimulating the migration of various cells, such as endothelial cells, at hypoxic and inflammatory tumor sites [74,75]. It upregulates Hypoxia-Inducible Factor-1α (HIF1α) expression, which increases VEGF expression and signals for angiogenesis [76]. The accumulation of IL6 also stimulates Metalloproteinase-9 (MMP9) production in OC, promoting cancer cell invasion and degradation of the extracellular basement membrane. MMP9 then releases and activates VEGF and the basic Fibroblast Growth Factor (FGF2), enabling endothelial cells’ growth and survival (Figure 2, Table 1) [77,78].

EMT is a physiologically controlled mechanism involved in wound healing, embryogenesis, and the development of ovaries [40]. Elevated levels of IL6 induce the expression and activation of various transcription factors responsible for EMT and promote invasive and metastatic traits of OC [23,79,80]. IL6 secreted by TAMs in OC-TME induces EMT by upregulating mesenchymal proteins, including Metalloproteinases, N-cadherin, Fibronectin, and Vimentin, and downregulating E-cadherin, Claudins, and Ocludins [23]. This leads to changes in the intercellular tight junctions and increased motility and leakage of cancer cells into the surroundings [23]. IL6 upregulates MMP2 and MMP9 in OC, which degrades the extracellular matrix and makes passages for tumor invasion and cancer cell metastasis [40]. Raised levels of MMP9 in OC indicate a poor prognosis and increased recurrence [81].

IL6-induced JAK/STAT, MAPK/ERK, PI3K/AKT, and NFκB pathways also lead to overexpression of EMT-transcription factors that hinder the production of E-cadherin at cell adherent junctions [82]. Loss of E-cadherin results in the rapture of cell-to-cell adhesion and subsequent detachment of the epithelial cells, thus increasing the invasiveness of OC cells from the tumor site [83]. E-cadherin downregulation is related to poor prognosis, as it provides metastatic seedlings of OC cells in the peritoneal cavity by upregulating expression of invasive adhesion molecules, including α5-integrin and CD44 that mediate OC cell adhesions to mesothelial cells in the abdominal cavity [83]. IL6-induced EMT leads to upregulation of Fibronectin and induction of OC invasiveness and metastasis [84,85,86]. IL6-mediated signaling pathways also downregulate endothelial tight junction proteins, such as Occludin and Claudin, weaken OC’s basement membrane, and increase invasiveness, migration, and chemoresistance (Figure 2, Table 1) [87,88]. Studies have shown that higher levels of IL6 promote ascitic fluid formation and are associated with poor outcomes in OC [89].

### 2.4. IL6 Induces Resistance to Treatment

Platinum (Cisplatin and Carboplatin) and Taxame (Paclitaxel) are the first-line chemotherapeutic drugs in OC. However, poor response or resistance to these drugs is frequently developed. IL6 via the JAK/STAT pathway has been shown to relate to the inadequate response of these chemotherapies in animal models [76]. CSC induced by IL6 also promotes therapeutic resistance [69,90]. Furthermore, high levels of IL6 and Tumor Necrosis Factor (TNF) in OC patients are associated with pretreatment ascites development, chemotherapeutic resistance, and shorter overall survival (Figure 2, Table 1) [91].

Immune checkpoints maintain immune homeostasis by regulating immune cells, which either activate or inhibit immune responses, ensuring proper function. Elevated IL6 levels activate signaling pathways and upregulate checkpoint molecules PDL1 and CTLA4 expression in cancer cells, which leads to inhibition of T cell activity and serves to escape immune response [41]. IL6-induced immune evasion leads to rapid proliferation, metastasis, and resistance to chemotherapy [92]. In OC, immune evasion and escape are crucial elements that correlate to poor survival outcomes (Figure 2, Table 1) [93].

## 3. IL6 as a Therapeutic Target for OC

### 3.1. IL6 Inhibitors

**Figure 3:**
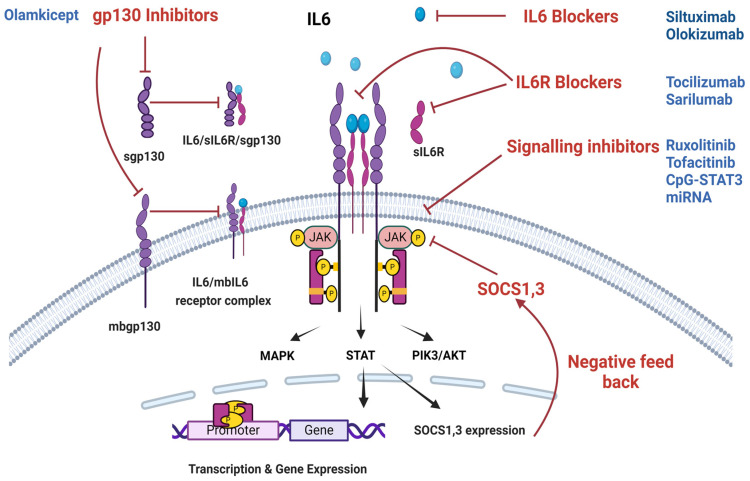
**Various IL6 pathway inhibitions.** IL6 inhibition at different parts of IL6 signaling pathways is effectively achieved by suppressing receptor activation, cellular signaling, and gene expression. Soluble IL6 receptor (sIL6R), membrane-bound IL6 receptor (mbIL6R), soluble Glycoprotein-130 (sGp130), membrane-bound Glycoprotein-130 (mbGp130), Mitogen-Activated Protein Kinase (MAPK), Signal Transducer and Activator of Transcription (STAT), Phosphatidylinositol-3 kinase (PI3K), Suppressor of Cytokine Signaling (SOCS).

IL6 inhibitors are monoclonal antibodies (mABs) that neutralize IL6 to inhibit its functional efficacy (Figure 3, Table 2) [33]. For instance, Siltuximab and a novel mAB, HZ-0408b, have shown better IL6 inhibition as an effective therapy in various diseases, including cancers [94,95,96,97]. IL6 inhibitors have been shown to prevent the conversion of non-stem cancer cells to cancer stem cells and alleviate cancer-associated anemia by increasing hemoglobin and reducing CRP levels. These benefits suggest a potential therapeutic value for IL6 inhibitors in ovarian cancer [65,98].

Siltuximab efficacy was investigated in a platinum-resistant Phase I OC clinical trial (NCT01637532), which showed decreased tumor growth and angiogenesis and diminished tumor macrophage infiltration (Table 3) [99]. In a Phase II clinical trial study with platinum-resistant OC, a well-tolerated therapeutic effect was observed, demonstrated by decreased VEGF and chemokines CCL2 and CCL12 levels in some patients (Table 3) [118]. Studies have also shown that anti-Il6 antibodies enhance Paclitaxel’s therapeutic efficacy and reduce platelet levels in OC mouse models [74]. Another high-affinity IL6 antibody, 1339, has exhibited promising results in various pre-clinical studies by inhibiting IL6 signaling pathways such as JAK/STAT, PI3/AKT, and MAPK/ERK pathways [119]. Recent studies have highlighted the potential of CNTO328, a chimeric murine anti-human IL6 antibody, to neutralize the function of IL6 and reduce the incidence of cancer-related anorexia and cachexia without serious adverse effects (Table 2) [100].

### 3.2. IL6R Inhibitors

**Figure 4:**
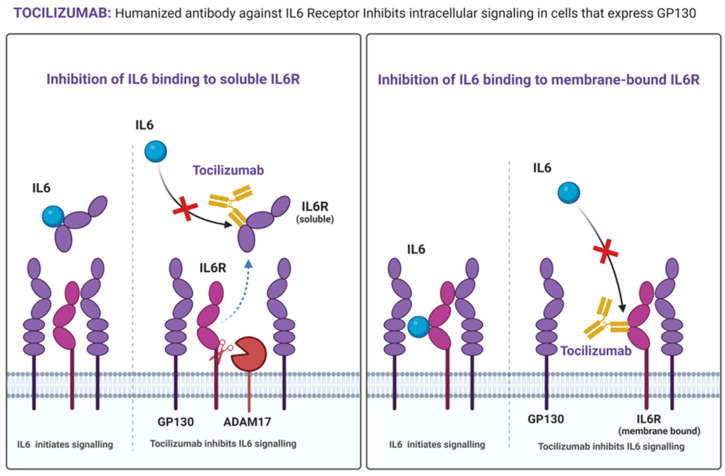
**Tocilizumab**. This monoclonal antibody recognizes soluble and membrane-bound IL6 receptors and inhibits IL6 signaling in cells. Interleukin 6 Receptor (IL6R), Glycoprotein-130 (Gp130), and A Disintegrin And Metalloproteinase-17 (ADAM17).

IL6 receptor (IL6R) inhibitors include monoclonal antibodies targeting IL6 receptors, such as Tocilizumab, Sarilumab, and Satralizumab (Figure 3 and Figure 4, Table 2) [137,138,139]. IL6R inhibitors, particularly Tocilizumab, have shown promise in managing cancer and various chronic diseases [138,139]. In OC, an increased expression of IL6 receptors is observed in the majority of OC cell lines (six out of seven, including RMUG-S, RMG1, OVISE, A2780, SKOV3ip1, and OVCAR3), indicating a heightened response to IL6 in these cells and increased responsiveness to anti-IL6R antibody therapy [140]. Additionally, anti-IL6R drugs have demonstrated their potential to resensitize chemotherapy-resistant cells, suggesting their incorporation into chemotherapeutic regimens could benefit them [140]. Increased IL6 production by platinum-based chemotherapy, Cisplatin/Carboplatin, promotes monocyte differentiation into M2 phenotype macrophages. Tocilizumab prevents M2 differentiation and may enhance the efficacy of platinum-based treatment (Table 3) [141].

### 3.3. Signaling Blockers

STAT3, a downstream IL6 effector, is activated in most cancers, including OC, and has been linked to aggressive OC clinical behavior [40]. Studies indicate that STAT3 contributes to the invasiveness of ovarian cancer by regulating cell motility through nuclei localization as well as focal adhesion, suggesting it is a potential target for therapeutic intervention [142]. STAT3-induced overexpression of tumor-promoting miR-216a downregulates tumor suppressor gene PTEN (phosphatase and tensin homolog), which regulates the PI3K/AKT (Phosphatidylinositol 3-kinase and Protein Kinase B) oncogenic pathway [143]. This dysregulation leads to increased OC cell proliferation, enhanced colony formation, and resistance to Cisplatin, the first-line therapy for OC [144,145,146,147,148]. Targeting STAT3 with Resveratrol, for example, blocks the downstream effects of IL6 and has shown potential in overcoming therapy-induced resistance in OC and various other cancers (Table 2) [105]. In OC cell lines (SKOV3), Cisplatin administration induces the secretion of CCL5 from TME, which activates STAT3 and PI3K/AKT signaling pathways, thereby suppressing the anti-apoptotic proteins Survivin and BCL2, and STAT3 inhibitors are potential targets for reversing resistance in patients (Table 3) [149,150]. Another study indicates that administering a STAT3 inhibitor reduces CD44 expression in biliary tract cancer and may be a promising target for reducing cancer cell stemness in OC [69,151]. Inhibition via STAT3-targeted knockdown of OC cells reduced the oncogenicity by decreasing CD44 and hTERT [71,152].

IL6/JAK/STAT signaling can activate PI3K/AKT pathways, which play a crucial role in cell metabolism, proliferation, and survival by activating AKT and mTOR (the mechanistic target of rapamycin), and is negatively regulated by *PTEN* [153]. The gene *PI3KCA* encodes the p11α catalytic subunit of PI3K (phosphatidylinositol 3-kinase), a key enzyme in the PI3K/AKT signaling pathway [154]. Genetic mutations in *PIK3CA* are commonly found in OC [155]. A Phase I/II clinical trial has indicated that targeted PI3K and AKT pathways could be beneficial, especially with specific genetic alteration, supporting targeting potential (Table 3) [106,107].

JAK inhibitors (JAKi) interfere with STAT3 phosphorylation and activation of JAK/STAT pathways. The Phase II trial of JAKi (Ruxolitinib) for metastatic pancreatic cancer showed improved patient survival (Table 3) [156]. Similarly, the Phase I/II study with Ruxolitinib shows improved survival in the experimental arm (14.6 months) as compared to the reference arm (11.6 months) [155]. Another OC study in mice found that combining a Jak2-specific inhibitor (CYT387) with Paclitaxel reduced the expression of OC cell biomarkers, including Oct4, Cd117, Ca125, and Ki67, in residual tumors and showed improved treatment outcomes [157].

### 3.4. IL6 Expression Blocker

NFκb inhibitors could lower IL6 expression at the transcriptional level in addition to STAT3 and JAK-STAT inhibition [158]. Bortezomib, an NFκb inhibitor, is primarily used to treat myeloma, which is known to produce high levels of IL6 [158]. Studies have explored its potential in other cancers, including OC. For example, a Phase II clinical trial evaluated the combination of Bortezomib with chemotherapy in patients with recurrent OC (Table 2) [159].

AT-Rich Interaction Domain-5α (ARID5α) inhibitors, such as Chlorpromazine, can act as a post-transcriptional blockage for IL6 by controlling IL6 production. They stabilize the mRNA of IL6, STAT3, and other related transcription factors and increase their expression [160]. ARID5α promotes tumor cell proliferation in various cancers, including breast, lung, pancreatic, colorectal, and glioma [161,162]. Thus, inhibition of ARID5α may be beneficial to combat OC (Table 2).

### 3.5. Tyrosine Kinase Inhibitors

In OC, proto-oncogene tyrosine-protein kinase (Src) activation has been associated with tumor progression, metastasis, and drug resistance. Src inhibitors, such as Dasatinib, Saracatinib, and Bosutinib, have been investigated for their potential in various cancer treatments [163,164]. Src has been shown to regulate IL6 signaling by activating downstream signaling pathways, such as the MAPK/ERK and NFκB pathways [165]. The Src homology-2 (SH2) is a protein domain on signaling proteins, including Src kinases and STAT proteins [166]. The interaction between the SH2 and phosphotyrosine motifs on the activated IL6R can initiate intracellular signaling cascades, leading to IL6-mediated cellular responses [166]. SH2 inhibitors are effective therapeutic agents for lowering IL6 signaling, but they are still being fundamentally considered for experimental and clinical trials (Table 2 and Table 3) [167].

### 3.6. Gp130 and sGp130 Inhibitors

Blocking the activity of Gp130 and soluble Gp130 (sGp130) shows promise as a potential drug for cancer (Figure 3, Table 2) [168,169]. SC144 is a Gp130 antagonist that targets Gp130 by increasing phosphorylation at Ser782 and downregulating Gp130 surface expression [108]. This thus inhibits downstream signaling pathways, including STAT3 and AKT [108]. Administration of SC144 has several effects on OC, including inhibition of tumor angiogenesis, increased apoptosis, enhanced cytotoxicity of tumor cells, and suppression of cell proliferation, cell cycle progression, and cell growth and survival [108]. The study suggests that targeting Gp130 with SC144 may be more effective than anti-IL6 antibodies in treating OC. Additionally, the study found that SC144 showed no toxicity to normal cells, further highlighting its potential as a targeted therapeutic agent [108].

### 3.7. RNAs and the Epigenetic Modifiers

Epigenetic modulations, including modification in DNA components and histones, telomer disruptions, expression of oncogenic and tumor suppressive microRNA (miR/miRNA), and non-long coding RNA, affect the regulation of IL6, IL6R, and its signaling. DNA methylation affects transcription sites of binding elements and thus alters gene expression. For instance, IL6 elevation in A549 lung cancer shows DNA hypermethylation via the JAK/STAT3 pathway, resulting in p53 and p51 downregulation and upregulation of DNA methyl transferase 1 (DNMT-1), leading to tumor progression [170].

miRNAs, a class of small non-coding RNAs, play a role in tumorigenesis by influencing the translation and degradation of mRNA. miRNA modulates epigenetically and disrupts IL6, IL6R, and its pathway, and their role has been observed in various cancers [171]. For instance, miR-182-5p significantly impacts the IL6 receptor complex, contributing to tumor aggravation by enhancing angiogenesis, apoptosis, and metastasis in ovarian, breast, colon, liver, and bladder cancers [172]. The tumor suppressor miR-34a is often reduced in some cancers, leading to decreased apoptosis and increased proliferation and metastasis. P53 induces transcription of miR-34a, and its mutation can lead to impaired expression. Additionally, epigenetic methylation, specifically inhibiting histone deacetylase in the promoter region of miR-34a, can cause its downregulation [173]. There is an inverse relationship between miR-34a and IL6 levels in various cancers, leading to increased expression of IL6R mRNA and IL6/STAT3 signaling, as observed in OC, colorectal, and pancreatic cancer [174,175,176].

In OC, upregulation of miR-21 is associated with the development of cancer cells, apoptotic escape, cell migration, and drug resistance [177]. Studies indicate that the miR-21 gene contains binding sites for STAT3, which, on IL6 induction, control its expression, whereas ectopically increased miR-21 in the absence of IL6 signals and STAT3 inhibition decreases the apoptotic process of cancer cells, showing the role of IL6-dependant miR-21 activation [178]. Another microRNA, miR-HOTTIP, has been found to increase IL6 expression in OC, which causes neutrophils to express PD-L1 and inhibit T cells and thus aid in immune escape in OC patients [179].

In OC, downregulation of tumor suppressor microRNAs, such as miR-125b and let-7, is related to tumor progression and predictive of therapy resistance [180,181,182,183,184,185]. Inhibition of IL6 signaling in cells overexpressing miR-125 and let-7e has been shown to reduce chemotherapeutic-induced IL6 elevation and improve sensitivity to Cisplatin [186].

IL6-activated mi-RNA down-regulates the expression of tumor suppressor ARH1 (Aplysia Ras homolog member 1), which inhibits cell migration and promotes autophagy in OC [187]. Studies indicate that IL6 negatively regulates autophagy by upregulating miRNAs such as hsa-miR-486-3p (target ULK2) and hsa-miR-21-5p (target ATG10), out of the six miRNAs that potentially target ARH1 [112]. Re-expression of ARH1 protein prevents IL6-induced Tyr705 STAT3 phosphorylation activation. IL6 also inhibits autophagosome LC3 (light chain 3) formation in OC cell lines, thus inhibiting cell mortality and autophagy and promoting cell migration [112].

IL6/STAT3 pathway-induced epigenetic changes can lead to autoimmune diseases. For instance, IL6 induces downregulation of RFX1 and leads to increased production and differentiation of Th-17 cells by decreasing DNA methylation and histone 3 lysine 9 (H3K9) trimethylation and increasing histone acetylation in patients with autoimmune disease SLE (systemic lupus erythematosus) and mice study model [188].

IL6 also induces epigenetic deregulation by histone modification in OC. Specifically, IL6 upregulates histone demethylase family JMJD2A (Jumonji C-domain family 2, also known as KDM4), which has higher expression than controls in OC cells [189]. Studies show that JMJD2A knockdown in OC cells inhibited IL6 expression, decreased proliferation, and enhanced sensitivity to Cisplatin [189]. Telomere length, which tends to shorten with repetitive cellular division in cancer cells, has been found to have an inverse relationship with IL6 levels in OC. This suggests that IL6 may play a role in telomere length dynamics and further cancer progression [190].

siRNA (small-interference RNA) is a small molecule of RNA (20–25 nucleotide in length) that can target mRNA molecules that are complementary in sequence to the siRNA, preventing it from being translated into protein and thereby reducing or silencing the expression of the targeted gene [191]. siRNA has been investigated for its therapeutic potential to reduce IL6 expression in vivo and in vitro. Targeting IL6 with siRNA sensitizes OC cells to chemotherapy and enhances chemotherapeutic effectiveness in patients [192]. As a novel therapy, siRNA can be identified as a promising way to address elevated IL6 levels in OC. The major challenges are siRNA instability, poor presentation, and off-target effects [193]. Innovations in delivery methods using nanoparticles are being researched to overcome these hurdles. Nanoparticle encapsulation and exosomes can improve their stability [194]. Chemical modifications such as 2’-O-methylation and advanced siRNA design can mitigate this issue [195].

Targeting epigenetic biomarkers such as miR-182-5p regulates IL6, its receptor complex, and signaling pathways, resulting in regression of tumor burden [173]. Similarly, replacement therapy with tumor suppressor miR-34a can downregulate IL6 expression and suppress the pro-inflammatory effects associated with IL6/STAT3 activation, thereby aiding tumor suppression and invasion control in OC [176].

Resveratrol is a naturally occurring polyphenol that can introduce epigenetic changes by micro-RNA modulation. Resveratrol could counteract the IL6 induction of cell migration in ovarian cancer cells through the induction of autophagy in the cells at the migration front, paralleled by the up-regulation of ARH1 and down-regulation of STAT3 expression [112].

### 3.8. Inhibition via Triterpenoid

The novel oleanolic acid derivative synthetic agents, Cyano-3,12-Dioxooleana-1,9(11)-dien-28-oic acid (CDDO) and its methyl ester CDDO-ME (C-28 Methyl, also known as Bardoxolone Methyl), can inhibit IL6 signaling and phosphorylation of STAT3, JAK2, and Src [196]. CDDO-Me increases sensitivity, decreases resistance to Paclitaxel chemotherapy, and increases apoptosis of cancer cells by reducing STAT3-induced expression of the anti-apoptotic genes in OC cell lines [116].

### 3.9. IL6 Inhibition Through Inhibition of Mitochondrial Fission

IL6 plays a pivotal role in ovarian cancer (OC) cells and promotes cancer progression by affecting mitochondrial dynamics. Mitochondrial fission is a process when a mitochondrion, the cell’s powerhouse organelle responsible for energy production, undergoes division into two or more smaller mitochondria [197]. This process is crucial for maintaining mitochondrial health and function, as it removes damaged or dysfunctional parts of the organelle and facilitates the distribution of mitochondria during cell division [197]. This process is regulated by dynamin-related protein-1 (Drp1) and other factors that respond to cellular signals and stressors (Table 2) [198]. Dysregulation of mitochondrial fission is associated with abnormal cell metabolism, proliferation, and metastasis, contributing to the aggressive nature of cancer [199].

Studies show that IL6 treatment increases metastasis in ovarian cancer cell lines (SKOV3 and PA1) by activating Drp1, a key mitochondrial fission regulator [116]. IL6 also triggered ERK1/2 activation, and blocking ERK1/2 reduced mitochondrial fission [200]. Inhibiting fission through siRNA or a pharmacological inhibitor significantly decreased IL6-induced migration and invasion, as shown in 3D invasion assays using patient-derived spheroids [200]. Inhibiting mitochondrial fission through genetic or pharmacological means such as Mdivi-1 significantly reduces IL6-induced migration and invasion of OC cells [200]. These findings suggest that targeting mitochondrial fission could help limit ovarian cancer metastasis.

## 4. Combining IL6-Targeted Therapy with Other Therapies

### 4.1. IL6 Co-Inhibition with Direct Inhibitors and/or Signal Blockers

Tocilizumab (Figure 4), the IL6R antibody, has been used in early-Phase clinical trials in OC and could suggest an improvement in immunological response when combined with chemotherapy (Table 3). Previous studies have shown promising results with Tocilizumab combination treatment in clinical trials for various inflammatory diseases, including cancers. For instance, rheumatoid arthritis has been managed traditionally with disease-modifying antirheumatic drugs (DMARDs) and methotrexate, but the co-administration of Tocilizumab substantially reduced morbidity in these patients [201]. In renal cancer carcinoma (RCC), combining Tocilizumab with tyrosine kinase inhibitors (TKI) inhibited angiogenesis and resulted in greater efficacy in suppressing tumor growth, suggesting a novel therapeutic approach for RCC [202].

The CD44/STAT3 axis is involved in cancer progression and therapy resistance. Combinatorial administration of the most promising targeting agents, such as A6-blocking peptide against CD44 and Napabucasin against STAT3, may effectively combat OC. IL6 depletion with STAT3 blockade has already been observed in liver cancer cells, where it enhanced apoptosis and reduced drug resistance [203]. STAT3 blockade via siRNA or specific inhibitors has significantly decreased CD44 expression in breast, prostate, nasopharyngeal, and gastric carcinoma models [142].

Combining Src inhibitors with JAK inhibitors, which contribute to IL6 inhibition, has improved apoptosis and inhibited cell proliferation in OC [204]. Similarly, studies show that IL6-induced chemoresistance is developed by cross-talk of MAPK/ERK and NFκB signaling; thus, upstream blockage of these pathways can potentiate the anti-tumor response of anti-IL6 and anti-IL6R antibodies [205].

IL6 overexpression has been identified as a significant contributor to chemoresistance in anti-EGFR therapy, suggesting that co-targeting of IL6 and Epidermal Growth Factor Receptor (EGFR) can be a beneficial and promising therapeutic approach for overcoming drug resistance [206]. Decreased cancer cell proliferative activity has been shown in non-small cell lung cancer and breast cancer when anti-IL6 antibodies were combined with EGFR small molecule inhibitor Erlotinib and anti-Human Epidermal Growth Factor Receptor (HER2) monoclonal antibody Trastuzumab, respectively [205]. Moreover, blockade of EGFR/ERK/NFκB and EGFR/PI3K/NFκB signaling not only decreases IL6 and IL6R-associated chemoresistance in OC but also, when combined with an anti-IL6 antibody (Tocilizumab), potentiates the anti-tumor effect [205].

### 4.2. IL6 Co-Inhibition with Interleukin I

IL1 drives IL6 production through NFκB and MAPK pathways in immune cells and fibroblasts, creating a feedback loop where IL6 amplifies IL1 activity, perpetuating chronic inflammation [207]. This IL1–IL6 cycle intensifies systemic inflammation (e.g., increasing C-reactive protein) and shapes a tumor-promoting environment by recruiting immune-suppressive cells and fostering angiogenesis, aiding tumor survival and immune evasion [208].

IL1 inhibitors, particularly Anakinra, Canakinumab, and MABp1 (Xilonix), have been explored in cancer treatment due to their anti-inflammatory effects [207]. Anakinra, an inflammasome recombinant IL1 receptor antagonist, blocks both IL1α and IL1β, showing potential in reducing tumor-related inflammation [209]. The inflammasome trans and IL-1β cytokines play critical roles in cardiovascular diseases like atherosclerosis, myocardial infarction (MI), myocarditis, and heart failure (HF) [210]. IL1 inhibitors, including Canakinumab (IL-1β antibody), Anakinra (recombinant IL1 receptor antagonist), and Rilonacept (IL1 receptor fusion protein), have shown promising results in clinical trials [210]. Canakinumab reduced the recurrence of ischemic events in patients with previous MI, while Anakinra improved outcomes in ST-segment elevation MI, HF with reduced ejection fraction, and recurrent pericarditis, where it is now the standard second-line treatment. Rilonacept has also shown potential for recurrent pericarditis [210]. Canakinumab, a monoclonal antibody targeting IL1β, demonstrated reduced cancer incidence in the CANTOS trial and reduced incidence and morbidity of lung cancer, spurring interest in its oncology applications [211]. MABp1, targeting IL1α, has shown promise in improving inflammation and cachexia in colorectal cancer [212].

In ovarian cancer specifically, preclinical studies and early trials with IL1 inhibitors like Anakinra suggest potential benefits in reducing the IL1–IL6 inflammatory loop, which promotes tumor growth and immune evasion. A study in ovarian cancer identifies IL1β as a key chemokine secreted by cancer cells that suppresses p53 expression in cancer-associated fibroblasts, fostering a pro-tumorigenic inflammatory microenvironment [212]. High IL1β and its receptor expression in cancer cells and CAFs correlate with poor patient survival [213]. These findings suggest that reduced secretion in TME IL1 and IL6 could help reduce tumor inflammation and growth, and combination drugs may be an effective therapy in OC

### 4.3. IL6 Inhibition Combined with Chemotherapy

A study carried out in chemo-resistant OC patients indicated that combining Tocilizumab with Carboplatin is more effective in inhibiting cell proliferation, offering a better therapeutic approach [205]. Platinum-resistant OC cells with increased resistance to Cisplatin exposure were shown to be mediated by increased IL6 and cellular Inhibitor of Apoptosis-2 (cIAP2) expressions [214]. The study suggests that co-targeting IL6 and cIAP2 with Cisplatin could offer a novel approach to treating drug-resistant OC [214].

These encouraging results provided a rationale for conducting pre-clinical and clinical trials that explore the combination of anti-IL6 monoclonal antibodies with existing chemotherapies for various types of cancers. A Phase I clinical trial in recurrent OC, with co-administration of anti-IL6R antibody, Carboplatin, Doxorubicin, and Interferon-α, showed no unexpected toxicity and less suppressive immune response and has also supported the progression to Phase II trials [99].

### 4.4. IL6 Inhibition Combined with Poly (ADR-Ribose) Polymerase (PARP) Inhibitors

PARP is a protein family mainly involved in DNA repair and genomic stability [215]. In 2014, PARP inhibitors were first approved for advanced OC with BRCA mutations [216]. The PARP inhibitors took away the DNA repair capacity of the BRCA mutant cells, thus allowing transformed BRCA mutant cells to undergo programmed cell death. PARP inhibitors were later shown to improve survival rates of women with OC with or without mutated BRCA [217,218]. In vivo, PARP inhibitors reduced the level of IL6 [219]. Combining IL6 inhibitor Bazedoxifene and PARP inhibitor Talazoparib was shown to synergistically reduce OC cell growth, suggesting that the combined treatment could provide better efficacy in treating OC [220].

### 4.5. IL6 Inhibition Combined with Estrogen Receptor Inhibition

Approximately 40–60% of OC express the estrogen receptor (ERα). IL6 activation of the MEK/ERK and PI3K/AKT pathways phosphorylates ERα, promoting drug resistance. Inhibiting IL6 expression may decrease ERα levels and counteract therapeutic resistance associated with ERα inhibition [221]. A dual approach of combining IL6 blockade with anti-estrogen hormonal therapy indeed arrested tumor growth [222].

### 4.6. IL6 Inhibition Combined with Checkpoint Inhibitors

A combination of anti-IL6 blockade and checkpoint inhibitors is promising to enhance immune response in cancer patients [223]. However, in OC, checkpoint inhibitors have resulted in poor survival. Combining checkpoint inhibitor therapy with drugs targeting specific pathways of IL6 might enhance efficacy, improve survival, and overcome cancer resistance [224]. In patients with non-small cell lung cancer, a lower level of baseline IL6 determines the response towards the PD1/PDL1 inhibition, suggesting that combining the two therapies will improve the effectiveness of fighting cancer [225].

### 4.7. IL6 Inhibition Combined with Naturally Occurring Components

Curcumin, a naturally occurring component in turmeric, is known to inhibit NFκb transcription and shows anti-tumorigenic and anti-angiogenic activity in chemo-sensitive and chemo-resistant OC cell lines [226]. Several other studies investigated the effects of Curcumin in OC and have shown that it enhances the effectiveness of chemotherapy, downregulates the chemotherapy resistance-associated protein Survivin, and inhibits the migration and invasion of OC cells, highlighting its potential as an adjunct therapy in OC treatment (Table 2) [227,228,229].

Quercetin, a plant-based flavonoid in various fruits and vegetables, presents a natural alternative with antioxidant and anti-cancerous potential. It impacts several cellular signaling pathways (Wnt/β-catenin, PI3K/AKT, JAK/STAT, MAPK, p53, NFκB) and regulates tumor-suppressive [230]. These pathways are IL6-activated, and various studies highlight Quercetin’s inhibitory effect on IL6 and subsequent aid in various cancers. For instance, Quercetin inhibited BPDE (benzopyrene diol epoxide, an active metabolite of cigarette smoke carcinogen)-induced IL6 secretion in lung fibroblasts via NFκB and ERK pathway suppression [231]. It also blocked IL6-induced STAT3 activation and prevented IL6-boosted HBEC (human bronchial epithelial cells) transformation [231]. In ovarian cancer, Quercetin demonstrates potent anticancer effects through various mechanisms, including induction of apoptosis, reduced metastasis, enhancement of chemosensitivity, radiosensitization, and overcoming Cisplatin resistance [232,233].

Quercetin’s low bioavailability limits its effectiveness; enhanced formulations like nanoparticles and phytosomes improve cellular delivery, while isoquercitrin offers a more bioavailable form with promising effects in cancers like kidney and pancreatic [112,230]. Ovarian cancer treatment with a Quercetin-loaded hydrogel system showed sustained release and effective anti-cancer activity, promoting apoptosis and inhibiting cell proliferation, highlighting its potential for chemotherapy [234]. Studies show that in nanoparticle formulations like PEGylated liposomal Quercetin (Lipo-Que), Quercetin can overcome Cisplatin resistance in ovarian cancer [235,236]. It induces cell cycle arrest and apoptosis in both Cisplatin-resistant (A2780cp) and Cisplatin-sensitive (A2780s) models, providing a potential strategy for treating resistant ovarian cancers [235].

Quercetin and Curcumin regulate ncRNAs, including miR-200b-3p, miR-21, and miR-30a-5p, influencing cell cycle, apoptosis, and cancer progression [237]. They also affect lncRNA pathways, offering potential therapeutic targets for cancer treatment [237].

Resveratrol is another naturally occurring polyphenol that can introduce epigenetic changes by micro-RNA modulation (Table 2) [113]. Resveratrol could counteract the IL6 induction of cell migration in ovarian cancer cells through the induction of autophagy in the cells at the migration front, paralleled by the up-regulation of ARH-I and down-regulation of STAT3 expression [112].

The RCQ, a compound combining resveratrol, Curcumin, and Quercetin, has shown enhanced anti-tumor effects in 4T1 breast cancer mice by boosting T cell recruitment, reducing immunosuppressive cell populations, and shifting the tumor microenvironment towards immune activation [238]. In vitro, RCQ increased ROS, reduced mitochondrial membrane potential, induced tumor cell apoptosis, and alleviated immunosuppression, enhancing anticancer effects [238]. These studies highlight the potential of these naturally occurring compounds, individually and preferably in combination, to inhibit IL6-activated pathways and may reduce the disease suffering related to ovarian cancer.

### 4.8. IL6 Inhibition Combined with OC-Specific Chimeric Antigen Receptor (CAR) T-Cell Therapy

CAR (Chimeric Antigen Receptor) T-cell therapy is one of the most robust T cell immunotherapies, successfully administered in various cancers, such as hematological tumors [239]. In OC, CAR-T-cell therapy is still maturing due to the typically associated hurdles with solid tumors, such as their diverse histopathology, immunosuppressive environment, tumor toxicity, finding specific antigens, or developing unfavorable side effects [240].

With CAR-T-cell therapy, there are possible side effects, including cytokine release syndrome (CRS) and neurotoxicity, and IL6 is known to be a significant factor in both events [241]. Co-administration of Tocilizumab can block IL6R in CRS but still allows the higher levels of IL6 to cross the blood-brain barrier and have the potential to impose life-threatening events in patients [241]. Another challenge is cost-effectiveness, as a substantial amount of Tocilizumab needs to be administered along with CAR-T-cell therapy. To overcome these, researchers engineered and incorporated an anti-IL6 single-chain variable fragment along with CAR-T cells so that it can automatically release along with the therapy to neutralize and suppress the IL6 storm, and this way, the neurotoxicity and efficacy of the therapy are also reserved [242,243].

## 5. IL6 Targeting in Ovarian Cancer Clinical Trials with Emerging Therapeutic Strategies

Recent clinical trials have explored the potential of IL6 inhibition, both as monotherapy and in combination with chemotherapy, to improve patient outcomes. The following table highlights the OC clinical trials in various Phases exploring the inhibition of the pathway that can eventually lead to IL6 inhibition and could aid in minimizing the aggressiveness of the disease (Table 3).

In trials investigating IL6 inhibitors, such as anti-IL6 mAB, Siltuximab effectively blocks the IL6 signaling pathways and IL6-induced gene expression. In Phase II clinical trials (MHRA 21313/0007), Siltuximab showed significant reductions in IL6, CCL2, CXCL12, and VEGF levels, leading to decreased angiogenesis and macrophage infiltration, further validating the potential of Siltuximab in altering the tumor microenvironment. However, in a dose-escalated study (NCT00841191, 2014), monotherapy with Siltuximab was well tolerated, but solid tumors such as OC and KRAS mutant cancer lacked activity, highlighting the need for combination therapies [244].

Similarly, Tocilizumab, an IL6 receptor inhibitor, was investigated in a Phase I/II clinical trial (NCT01637532) with Carboplatin and pegylated liposomal doxorubicin. The result showed that the IL6 functional blockade was well-tolerated and suggested the potential for combination regimes in OC [99].

The significance of IL6 inhibition is particularly pronounced when combined with other targeted therapies. Various clinical trials have evaluated IL6 down signaling pathway inhibition with combination therapies to show enhanced therapy efficacy. Ruxolitinib, a JAK1/2, was combined with Carboplatin and Paclitaxel in a clinical trial (NCT02713386, 2021), showing prolongation of progression-free survival (PFS), emphasizing the value of IL6 pathway modulation in improving outcomes [120].

Additionally, studies have explored the effect of tyrosine kinase inhibitors in combination with traditional OC chemotherapy, particularly in resistant patients. Sunitinib, a tyrosine kinase inhibitor, was integrated with Carboplatin, Paclitaxel, and Doxorubicin (ACCR-17(8); 2011), which highlighted the overexpression of the IL6-STAT3-HIF pathway in ovarian clear cell carcinoma [26]. Sunitinib showed a therapeutic response in patients, particularly in addressing associated complications such as hypercalcemia and thromboembolism. Other tyrosine kinase inhibitors, such as imatinib and nintedanib, have also been studied in clinical trials and may offer promising therapeutic options for OC when combined with other agents [245].

Clinical trials with Pictilisib (a pan-PI3K inhibitor) and MK-2206 (an AKT inhibitor) have shown some promising results in OC with specific molecular alteration such as *PIK3CA* mutation or PTEN loss [106,154]. In Phase I trials, both drugs showed some clinical activity, with partial responses and improvements in tumor markers like CA125, particularly in molecularly selected patients [154]. However, Phase II trials revealed modest tumor shrinkage, indicating that their effectiveness is likely restricted to a molecularly defined subset [106]. These results suggest a need for personalized treatment approaches and combination therapies to improve clinical outcomes. The combination of MK-0752 and Tocilizumab significantly decreases BCSCs and inhibits tumor growth and thus might serve as a novel therapeutic strategy for treating women with Notch3-expressing breast cancers [246].

Cediranib, a VEGFR-TKI, has demonstrated promise in ovarian cancer (OC) trials, mainly when used in combination therapies. When paired with Olaparib, a PARP inhibitor, trials such as NCT01116648 have shown enhanced efficacy, improving outcomes in patients with recurrent or advanced OC [247]. Similarly, combinations with chemotherapy agents like Carboplatin and Paclitaxel (e.g., NCT00275028) have led to improved progression-free survival (PFS) and were generally well tolerated. Importantly, high baseline IL6 levels are associated with poorer outcomes, suggesting that Cediranib’s anti-angiogenic properties may help modulate IL6-related pathways, potentially enhancing therapeutic responses and addressing inflammation-driven tumor progression.

Recombinant human IL6 (rhuIL6) was used in clinical trials for ovarian cancer patients. A Phase Ib trial demonstrated its safety and ability to accelerate platelet recovery with Carboplatin and Paclitaxel [135]. However, later, a Phase II randomized trial, combining rhuIL6 with G-CSF, showed minimal thrombopoiesis effects in the same chemotherapy context, indicating limited benefit [136].

Recent ongoing Phase I/II trials investigating nanoparticle-encapsulating a Stat3/NFκB/poly-tyrosine kinase inhibitor with low-dose doxorubicin may mark a new era of efficient ways of IL6 inhibition in OC patients. The findings suggest that while IL6 inhibition alone may have limited standalone clinical efficacy, combining it with other therapeutic agents, particularly chemotherapies or pathway inhibitors, holds significant promise for improving outcomes in ovarian cancer patient.

## 6. Conclusions and Future Perspectives

IL6 at high levels in the TME of OC promotes disease progression, immune evasion, metastasis, resistance to treatment, and poor survival outcomes. In the earlier course of the disease, inflammation promotes cancer growth, while in advanced stages, inflammation promotes tumor spread and therapy resistance. Therefore, it would be beneficial to support effective disease management and improve the survival outcomes in OC patients.

Various clinical trials have provided valuable insight into the potential of IL6 inhibitors, such as Tocilizumab and Siltuximab, and pathway modulators like JAK, STAT, and tyrosine kinase inhibitors in managing OC. While monotherapy has demonstrated safety, limited efficacy underlines the necessity of innovative combinations. Emerging advancements in modes of drug delivery, such as nanoparticles encapsulated, fusion proteins, epigenetic modification, or even understanding the use of naturally available IL6 inhibitors, opened avenues for precision oncology tailored to individual patient needs.

Further research and trials should focus on exploring IL6 blockade in combination with existing therapies, leveraging modern innovations. Such integrative and comprehensive approaches can enhance therapeutic efficacy and improve overall survival. Addressing inflammation as a cornerstone of OC management, IL6 blockades with various available therapies may provide robust, patient-centered care, promote promising responses, and significantly reduce OC patients’ morbidity and mortality rates.

## Figures and Tables

**Table 1:** Contributions of IL6 in OC.

OC Processes	IL6 Action	Signaling Involved	Ref.
Proliferation induction	Upregulates the cell cycle-promoting proteins and downregulates cell cycle inhibitors	JAK/STAT and NFκB	[23]
Apoptosis inhibition	Upregulates antiapoptotic genes	JAK/STAT, MAPK/ERK, PI3K/AKT, and NFκB	[24]
Cancer stem cell generation	Promotes expression of CD44 and converts cancer cells to cancer stem cells	IL6/STAT3	[25]
Angiogenesis	Upregulates proangiogenic factors and stimulates endothelial cell migration	IL6/STAT3/HIF	[26]
Metastasis	Induces EMT, degrades extracellular matrix, and induces Fibronectin	JAK/STAT, MAPK/ERK, PI3K/AKT, EGFR, and NFκB	[27]
Malignant ascites formation	High IL6 levels in the ascitic fluids correlate with poorer outcomes	IL6/STAT3 and MAPK/ERK	[28]
Resistance to the first line of treatment	Apoptosis inhibition, induction of proliferation, and cancer stem cell formation	IL6/STAT3 and AKT	[29]
Immune escape	Upregulate checkpoint molecules, recruit immune suppressor cells and impair the activity of anti-tumor immune cells	JAK/STAT, MAPK/ERK, PI3K/AKT, and NFκB	[30]
Neoantigen expression	Upregulates enzyme to initiate NMD (nonsense mRNA decay)	IL6/STAT3	[31]
Anaerobic glycolysis	Inhibits pyruvate dehydrogenase activity by upregulating pyruvate kinase	IL6/STAT3	[32]

IL6: Interleukin-6, STAT3: Signal Transducer and Activator of Transcription-3, HIF: Hypoxia Inducible Factor, JAK: Janus Kinase, MAPK: Mitogen-Activated Protein Kinase, ERK: Extracellular Signal-Regulated Kinase, PI3K: Phosphoinositide-3 Kinase, AKT: Protein Kinase B, EGFR: Epidermal Growth Factor Receptor, NFκB: Nuclear Factor kappa light chain enhancer of activated B cells.

**Table 2:** Various strategies to target IL6.

IL6 Inhibition	Target	Mode of Action	Examples	Ref.
Direct inhibitors	IL6	Antibodies that neutralize IL6 and prevent its interaction with the IL6 receptor	Siltuximab, Olokizumab, and CNTO328	[99,100]
Receptor inhibitors	IL6R	Antibodies block the IL6 receptor	Tocilizumab, Sarilumab, and Satralizumab	[101]
Expression blockers	NFκB and ARID5α	Molecules that block the protein activities	Bortezomib, Curcumin, and Chlorpromazine	[102,103]
IL6 Signaling	JAK and STAT3	Molecules inhibit downstream signal	Ruxolitinib	[104]
STAT3 inhibitor	STAT3	Signaling blockers	siRNA-PLGA/CSO, and oncolytic adenovirus (M4)	[105]
PI3k inhibitors	PI3k	Signaling blockers	Pictilisib	[106]
AKT inhibitors	AKT	Signaling blockers	MK-2206	[107]
Gp130 inhibitors: IL6 signaling blockers	Gp130 or sGp130	Molecules that block the protein activities	SC144	[108]
Tyrosine kinase Inhibitors	EGFR/HER/ErbB family	Molecules inhibit IL6 signaling	Cetuximab, Gefitinib, and Lapatinib	[109]
Tyrosine kinase Inhibitors	Src kinase family	Molecules blocking Src activity, signaling blockers	Dasatinib, Saracatinib, and Bosutinib	[110,111]
Epigenetic modifier	Various miRNAs, lncRNA HOTTIP, DNMT1, and JMJD2A	Molecules targeting epigenetic protein, DNA methylation, Histone modification, and specific RNA to reduce IL6 signaling	Resveratrol, Decitabin, Azactadine, Guadicitabine, Belinostat, and Vorinostat	[112,113,114]
Mitochondrial fission inhibitors: IL6 signaling blockers	Drp1	Small molecules targeting Drp1 to reduce mitochondrial fragmentation	Mdivi-1	[115]
Natural inhibitors	Wnt/β-catenin, PI3K/AKT, JAK/STAT, MAPK, p53, and NFκB	Natural compounds targeting pathways involved in IL6 signaling	Curcumin, Quercetin, and Resveratrol	[112]
Synthetic triterpenoid and Inhibitors	STAT3, JAK2, and Src	Synthetic compounds targeting pathways involved in IL6 signaling	CDDO-Me	[116]
IL1 inhibitors	IL1	Antibodies or molecules that block IL1, indirectly affecting IL6 pathways	Anakinra, Canakinumab, MABp1 (Xilonix), and Rilonacept	[117]

IL6: Interleukin 6, IL6R: Interleukin 6 Receptor, NFκB: Nuclear Factor kappa-light-chain-enhancer of activated B cells, ARID5α: AT-Rich Interaction Domain 5A, JAK: Janus Kinase, STAT3: Signal Transducer and Activator of Transcription 3, PLGA/CSO: Poly(lactic-co-glycolic acid)/Chitosan, Gp130: Interleukin 6 signaling protein, EGFR: Epidermal Growth Factor Receptor, HER: Human Epidermal Growth Factor Receptor, ErbB: Erb-B2 Receptor tyrosine kinase family, Src: Proto-oncogene tyrosine-protein kinase Src, miRNAs: MicroRNAs, lncRNA: Long Non-Coding RNA, HOTTIP: HOXA Transcript at the Distal Tip, DNMT1: DNA Methyltransferase 1, JMJD2A: Jumonji Domain Containing 2A, Drp1: Dynamin-related protein 1, PI3K: Phosphoinositide 3-Kinase, AKT: Protein Kinase B, MAPK: Mitogen-Activated Protein Kinase, p53: Tumor Protein p53, CDDO-Me: 2-Cyano-3,12-dioxooleana-1,9(11)-dien-28-oic acid methyl ester.

**Table 3:** Ovarian cancer clinical trials targeting IL6 and its pathways.

Inhibitor	Study	Drug	Phase	Combination Drugs	Details	Status
**IL6 Inhibitor**	NCT01637532 [99]	Tocilizumab (IL6R inhibitor)	I/II	Carboplatin and pegylated liposomal doxorubicin (PLD)	Functional IL6R blocking is feasible and safe in EOC patients	Completed
	NCT00841191 [120]	Siltuximab (IL6 inhibitor)	I/II	Carboplatin and Paclitexel	Siltuximab showed good safety but limited efficacy as monotherapy in advanced OC.	Completed
	MHRA 21313/0007 [118]	Siltuximab	II	-	Significant reduction in IL6, CCL2, CXCL12, and VEGF leads to inhibited production of cytokines, reduction in angiogenesis, and less infiltration of macrophages.	Completed
**Tyrosine Kinase Inhibitor**	NCT01669798 [121]	Nintedanib (TKI)	II	Bevacizumab	Biomarker data reveal a prognostic association between high baseline IL6 levels and worse survival outcomes. This is consistent with our recent study of IL6 in chemotherapy in naïve, newly diagnosed OC.	Completed
	NCT00543049 NCT00979992 NCT01824615 NCT00388037 NCT00768144 NCT00453310 NCT00478426 NCT00474994	Sunitinib (TKI)	II	-	Evaluating the efficacy of the drug in OC	Completed
	Potential Target [26]	Sunitinib (TKI)	-	Carboplatin, Paclitaxel, and Doxorubicin	The IL6-STAT3-HIF pathway is overexpressed in OCCA and linked to hypercalcemia, thromboembolism, and potential therapeutic targets like MET, with responses to sunitinib being observed	Completed
	NCT02867956 NCT03075462	Apatinib (TKI)	II I	Etoposide Fluzoparib	Evaluating the efficacy of the drug in OC	Completed
	NCT01608009 NCT01238770 NCT01227928 NCT00281632 NCT02383251 NCT00866697 NCT01644825 NCT01262014 NCT01610206 NCT00561795 NCT01402271 NCT01468909 NCT02009449	Pazopanib (GW78603) (TKI)	I I/II II II II III II II II II I/II II I	Paclitaxel Cyclophosphamide Placebo - Paclitaxel Placebo Paclitaxel - Gemcitabine Carbo/pacli Carbo/pacli Paclitaxel Multiple	-	Completed
	NCT02055690 NCT01035658	Pazopanib	I/II	Fosbretabulin Doxil	-	Terminated
**Ras/Raf/MEK/ERK Pathway**	NCT00436215 [122]	Sorafenib	II	Bevacizumab	The combination showed some activity in platinum resistance OC cases. No difference in IL6 was identified.	Completed
	NCT00791778 NCT01047891 NCT00096395 NCT00093626 NCT00390611 NCT00096200		II	Plecebo Topotecan Gemcitabine - Carbo/Pacli Carbo/Pacli	Evaluating the efficacy of the drug in OC in combination with various drugs	Completed
	NCT00526799 NCT00522301		I/II II	Topotecan In remission		Terminated
**c-Met receptor TKI**	NCT01178411	Tivantinib (ARQ-197)	I/II	Anti-cancer chemotherapy	Evaluating the efficacy of the drug in OC in combination with various drugs	Completed
**Vascular endothelial growth factor (VEGF) receptor TKI (RTK)**	NCT01116648 NCT02446600 NCT02345265 NCT02502266 NCT03660826 NCT04739800	Cediranib	I/II III II II/III II II	Olaparib	Evaluating the efficacy of the drug in OC in combination with various drugs	Active
**Vascular endothelial growth factor (VEGF) receptor TKI (RTK)**	NCT00275028 NCT02340611 NCT02681237 NCT03117933 NCT00475956 NCT02855697 NCT01065662 NCT02889900 NCT00278343 NCT01131234	Cediranib (AZD2171)	II II N/A II I I I II II I	Olaparib and Chemotherapy	Evaluating the efficacy of the drug in OC in combination with various drugs	Completed
**Antiangiogenic**	NCT00262847 [123]	Bevacizumab	III	Carboplatin and Paclitaxel	OC patients in GOD-0218 trial benefited from the addition of Bevacizumab	Completed
**JAK 1/2**	NCT02713386 [120]	Ruxolitinib	I/II	Carboplatin and Paclitaxel	Well tolerated with acceptable toxicity in combination with chemotherapy	Active
**PI3K/AKT/mTOR Pathway**	NCT00960960 [106]	Pictilisib (GDC-0941) (Pan-PI3K inhibitor)	I	Paclitaxel, Bevacizumab, And Letrozole	Reduce tumor metabolism and growth in cases of *PIK3CA* amplification and *PTEN* loss	Completed
	NCT01283035 [107]	MK-2206 (AKT inhibitor)	II	Platinum resistance, *PI3KCA* and AKT mutations, and *PTEN* loss	Well-tolerated (skin rash) and showed disease stability	Completed
	NCT01653912 [124]	Afureserti/ GSK2110183 (AKT inhibitor)	I	Carboplatin, Paclitexel	Stable and partial response in recurrent platinum-resistant EOC	Completed
	NCT00431054 [125]	Perifosine (PI3K/AKT inhibitor)	I	Docetaxel	Combination therapy was well tolerated, with some patients expressing a stable and partial response	Completed
	NCT01031381 [126]	RAD001 (mTOR inhibitor)	II	Bevacizumab (VEGF inhibition)	Combination therapy was well tolerated	Completed
	NCT00886691 [127]	Everolimus (mTOR inhibitor)	II	Bevacizumab (VEGF inhibition)	PFS was not prolonged with a combination therapy	Completed
	NCT00586443 [128]	Everolimus (mTOR inhibitor)	I	Bevacizumab, Panitumumab (VEGF, EGFR inhibition	Prolonged disease control achieved in OC patients	Completed
	NCT01708161	Alpelisib (BYL719	Ib/II	Ganitumab (AMG 479)	-	Terminated
	NCT01197170 [129]	Everolimus (RAD001) (mTOR inhibitor), Sorafenib (BAY43-9006) (multikinase inhibitor), Erlotinib (OSI-774) (EGFR inhibitor)	I	Bevacizumab Anastrozole and Fulvestrant (anti-estrogenic)	Everolimus (RAD001) and Anastrozole were well-tolerated in patients with molecular alterations, *PIK3CA* mutations, and *PTEN* loss	Completed
	NCT01243762 [130]	MK-2206 (AKT inhibitor) rodaforolimus (mTOR inhibitor), MK-0752 (NOTCH inhibitor)	I	Dalotuzumab (IGF-1R inhibitor)	Combination therapy was tolerable	Terminated
**STAT3**	NCT02417753 [131]	AZD9150	-	-	Antisense oligonucleotide in malignant ascites	Terminated
	NCT03382340 [132]	IMX-110	I/II	Nanoparticles encapsulate a Stat3/NFκB/poly-tyrosine kinase inhibitor and low-dose doxorubicin	-	Active
	NCT00955812 [133]	OPB-31121	I	-	Inconsistent absorption and low bioavailability limit clinical utility	
**RANKL** **Inhibitor**	NCT03382574 [134]	Denosumab	I	-	-	Terminated
**TLR-7**	NCT00319748	853A	II	-	IL1ra	Completed
**rhuIL6**	IL6 toxicity in OC [135]	Recombinant human Interleukin-6 (hematopoietic stimulation- thrombopoiesis)	Ib	Carboplatin and Paclitaxel	IL6 is a well-tolerated cytokine that accelerates platelet recovery in chemotherapy patients.	Completed
	Advanced EOC [136]		II	G-CSF, Carboplatin, and Paclitaxel	Minimal thrombopoiesis effect in women OC with Paclitaxel–Carboplatin therapy	Completed
**IL6**	NCT00460200	IL6	-	-	To study depression and IL6 in EOC	Withdrawn

Clinal trials outcomes and related information from published articles: ClinicalTrials.gov. NCT: National clinical trial. I, II, II: Phases of clinical trial, OC: ovarian cancer, EOC:Epithelial ovarian cancer, PFS: progression free survival, IL6 Inhibitor: Interleukin-6 Inhibitor, IL6R Inhibitor: Interleukin-6 Receptor Inhibitor, IL6-STAT3-HIF: Interleukin 6-Signal Transducer and Activator of Transcription 3-hypoxia induced factor, TKI: tyrosine kinase inhibitor, c-Met Receptor TKI: c-Met Tyrosine Kinase Inhibitor, VEGF Receptor TKI (RTK): Vascular Endothelial Growth Factor Receptor Tyrosine Kinase Inhibitor, JAK 1/2 Inhibitor: Janus Kinase 1/2 Inhibitor, STAT3 Inhibitor: Signal Transducer and Activator of Transcription 3 Inhibitor, PI3K Inhibitor: Phosphoinositide 3-Kinase Inhibitor, AKT Inhibitor: Protein Kinase B (AKT) Inhibitor, mTOR Inhibitor: Mammalian Target of Rapamycin Inhibitor, RAF Inhibitor: Rapidly Accelerated Fibrosarcoma Kinase Inhibitor, MEK Inhibitor: Mitogen-Activated Protein Kinase Kinase Inhibitor, ERK Inhibitor: Extracellular Signal-Regulated Kinase Inhibitor, NFκB Inhibitor: Nuclear Factor kappa-light-chain-enhancer of activated B cell inhibitor, RANKL Inhibitor: Receptor Activator of Nuclear Factor kappa-Β ligand inhibitor, EGFR Inhibitor: Epidermal Growth Factor Receptor Inhibitor, TLR7 Inhibitor: Toll-Like Receptor 7 Inhibitor.

## Data Availability

No new data were created or analyzed in this study. Data sharing is not applicable to this article. Data for clinical trials in the table were collected from online databases for clinical trials: ClinicalTrials.gov. All figures were created using Biorender.com (https://BioRender.com).
